# Antifouling (Bio)materials for Electrochemical (Bio)sensing

**DOI:** 10.3390/ijms20020423

**Published:** 2019-01-19

**Authors:** Susana Campuzano, María Pedrero, Paloma Yáñez-Sedeño, José M. Pingarrón

**Affiliations:** Departamento de Química Analítica, Facultad de CC. Químicas, Universidad Complutense de Madrid, E-28040 Madrid, Spain; mpedrero@quim.ucm.es (M.P.); yseo@quim.ucm.es (P.Y.-S.)

**Keywords:** (Bio)fouling, electrodes, (bio)materials, complex biofluids, polymers, hydrogels, peptides, thiolated self-assembled monolayers

## Abstract

(Bio)fouling processes arising from nonspecific adsorption of biological materials (mainly proteins but also cells and oligonucleotides), reaction products of neurotransmitters oxidation, and precipitation/polymerization of phenolic compounds, have detrimental effects on reliable electrochemical (bio)sensing of relevant analytes and markers either directly or after prolonged incubation in rich-proteins samples or at extreme pH values. Therefore, the design of antifouling (bio)sensing interfaces capable to minimize these undesired processes is a substantial outstanding challenge in electrochemical biosensing. For this purpose, efficient antifouling strategies involving the use of carbon materials, metallic nanoparticles, catalytic redox couples, nanoporous electrodes, electrochemical activation, and (bio)materials have been proposed so far. In this article, biomaterial-based strategies involving polymers, hydrogels, peptides, and thiolated self-assembled monolayers are reviewed and critically discussed. The reported strategies have been shown to be successful to overcome (bio)fouling in a diverse range of relevant practical applications. We highlight recent examples for the reliable sensing of particularly fouling analytes and direct/continuous operation in complex biofluids or harsh environments. Opportunities, unmet challenges, and future prospects in this field are also pointed out.

## 1. Introduction

Despite the huge progress demonstrated by electrochemical biosensors for individual and/or multiplexed determination of a wide variety of relevant analytes including biomarkers at different molecular levels, the determination of certain compounds such as phenols and neurotransmitters [1,2], the direct determination of (bio)markers in complex media, and the continuous operation in biological matrices remain as important challenges due to the occurrence of (bio)fouling. This process is associated with gradual passivation of the transducer surface due to accumulation of fouling compounds which may be a matrix component, the target analyte, or an electrochemical reaction product [2]. Electrode (bio)fouling occurs by a broad range of mechanisms which depend mostly on the fouling agent type. It generally involves nonspecific adsorption and polymerization or precipitation of fouling agents including phenols, amino acids, neurotransmitters, proteins, and other biomolecules, such as cells (whole or fragments) and DNAs or RNAs, which form an increasingly impermeable layer on the electrode. Fouling agents tend to adhere to the electrode surface through different interactions (hydrophobic, hydrophilic, and electrostatic) depending on the type of fouling agent and electrode surface. Soluble proteins are relevant fouling agents because they possess dual behavior (hydrophilic on the surface to interact with an aqueous environment and hydrophobic on the inside to maintain protein folding) and, therefore, can foul electrodes through both interactions types. Regarding important analytes that foul electrodes, the electrochemical oxidation of phenols forms radicals and therefore polymeric structures which are insoluble and precipitate from solution on the electrode surface. On the other hand, the reaction products of neurotransmitters oxidation form polymeric molecules similar to melanin able to form both strong covalent bonds and noncovalent bonds with organic moieties and inorganic groups, respectively, of the electrode surface. All these phenomena inhibit the direct contact of the target analyte with the electrode surface for electron transfer to elicit an electrochemical response and severely affects the analytical characteristics of the corresponding (bio)sensors including sensitivity, reproducibility, stability, and overall reliability [2,3]. 

Therefore, the development of (bio)sensing interfaces that combine high sensitivity and antifouling ability by preventing byproducts, macromolecules and polymeric substrates from absorbing onto the electrode surface is essential for expanding the practical applicability of biosensors to allow reliable measurements [4,5].

It is worth noting that the exceedingly broad types of fouling agents and the ways in which they foul electrodes make equally broad the strategies developed to impart fouling resistance to the electrochemical sensors [2]. However, most antifouling strategies involve the use of a modified electrode with improved fouling resistance than the nonmodified electrode. Efficient efforts to minimize surface (bio)fouling have focused mainly on constructing antifouling biosensing electrochemical interfaces by using materials such as polymers, hydrogels, peptides, and thiolated self-assembled monolayers (SAMs) [5,6]. 

The following sections review the most relevant aspects of the main strategies involving biomaterials reported so far to develop electrochemical biosensors with excellent antifouling properties, capable to be employed in the analysis of undiluted or scarcely diluted complex samples and/or to provide excellent performance after prolonged incubation in raw biological fluids and in extreme pH media. Other nonfouling strategies involving the use of carbon materials, metallic nanoparticles, catalytic redox couples, nanoporous electrodes and electrochemical activation have been reviewed previously [2] and are out of the scope of this review. In addition to representative examples of each strategy, the main challenges to be addressed and future prospects for reducing fouling in electrochemical sensing and improve practical operation in complex environments are also briefly discussed.

## 2. Antibiofouling Polymers

Nonspecific protein adsorption can be minimized by modifying electrode surfaces with hydrophilic, conducting, zwitterionic, and pH-responsive methacrylate polymers. While hydrophilic polymers avoid biofouling by forming a hydration layer, hydrophobic surfaces prevent it by releasing the adsorbed proteins and cells [7,8].

The most commonly employed polymer to date for this purpose is poly (ethylene glycol) (PEG)-containing polymers and reagents, because of their commercial availability [9]. Recently, emerging zwitterionic polymers have shown to form stronger hydration layers and to be less prone to auto-oxidation in many biochemically relevant solutions [10,11,12]. In addition, they show advantages for in vivo applications in terms of biodegradability and low immunogenicity compared to the conventional hydrophilic PEG [13,14]. 

As for the polymer composition employed and since many conjugation chemistries required for chemical immobilization of biomolecules affect negatively the stability and/or function of the polymer [15,16], thin films combining surface attachment, antifouling and targeting agent sequentially controlled binding “motifs” are highly desirable [14].

### 2.1. PEG Polymers

PEG is a nontoxic and highly hydrophilic biocompatible polymer. It is considered the “gold standard” of antibiofouling polymers and has been widely used to reduce nonspecific protein adsorption [3]. Surface packing density and polymer chain length strongly affect the PEG antifouling properties [8]. A surface can be functionalized with PEG using two different approaches: by adsorbing a presynthesized PEG onto the surface or by growing in situ the polymer via a surface adsorbed initiation group [17]. Both strategies confer surfaces with protein and cell fouling resistance [7]. In general, it has been claimed that a benefit of using long polymer chains is related to a more efficient surface coverage. In this context, better surface coverages can be obtained by the spontaneous self-assembly of complex structures starting from separated PEG molecules.

The protein resistance of long-chain PEG modified interfaces is attributed to both hydration and steric hindrance effects [3,8]. Each ethylene glycol unit in the PEG backbone can strongly bind to one water molecule, bridging the ether oxygen along the 72 helical PEG chain [18,19,20], thus, resulting in the formation of a highly hydrated layer [8]. The compression occurring of this layer when a protein approaches the hydration layer barrier, leads to the biomolecule repulsion [7]. Despite the attractiveness of PEG as antifouling agent, its antifouling capabilities over long-term applications is limited by the low surface densities and susceptibility to oxidative damages [7,21,22,23]. Moreover, grafting of long chain antifouling nonconductive polymers, such PEG, can lead to the formation of layers with high impedance and result in a decrease in the electrode sensitivity. Accordingly, the incorporation of PEG with certain conductive soft materials such as conducting polymers has demonstrated to be helpful to address this problem and develop low fouling but highly sensitive assays [3]. 

**Figure 1:**
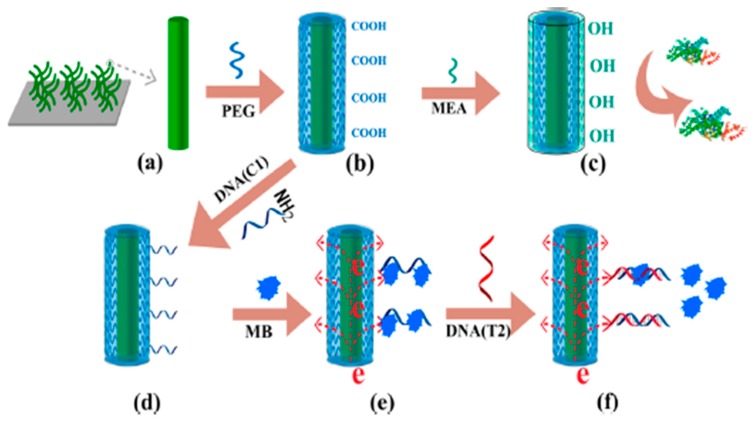
Steps involved in the preparation of an electrochemical nucleic-acid biosensor for *BRCA1* determination fabricated onto a PEGylated polyaniline glassy carbon electrode (PANI/PEG/GCE). (**a**) Deposition of PANI nanofibers on the GCE; (**b**) PEG modification onto PANI nanofibers; (**c**) monoethanolamine (MEA) modification; (**d**) immobilization of DNA capture probe (C1) onto PANI/PEG/GCE; (**e**) MB interaction with C1; and (**f**) hybridization with target DNA (T2). Reproduced from [3] with permission.

Hui et al. [3] reported the use of PEGylated polyaniline (PANI/PEG) nanofibers, prepared by grafting of PEG polymer onto PANI nanofibers, to avoid fouling in electrochemical nucleic acid biosensors prepared for the determination of the breast cancer susceptibility gene (*BRCA1*) in human serum. The biosensor involved immobilization of specific capture DNA probe onto a glassy carbon electrode (GCE) modified with the (PANI/PEG) nanofibers, a direct hybridization assay, and differential pulse voltammetric (DPV) monitoring of the decrease in the methylene blue (MB) signal after the hybridization with the target DNA (Figure 1). The DNA sensor showed a linear range from 0.01 pM to 1 nM with a detection limit (LOD) of 0.0038 pM, as well as satisfactory selectivity to discriminate DNA mismatches, and was successfully applied to the analysis of serum samples from breast cancer patients and healthy controls. Interestingly, the biosensor retained 92.17% of the initial current after incubation in undiluted human serum.

### 2.2. Conducting Polymers

Conducting polymers, with high electronic conductivity and porosity [24], have attracted considerable attention for preventing electrode fouling [25,26,27,28]. 

Yang et al. [1] proposed the use of poly (3,4-ethylenedioxythiophene)-poly (styrene sulfonate) (PEDOT:PSS) as a GCE antifouling modifier to prepare an electrochemical sensor for continuous monitoring of gaseous tricresyl phosphate (TCP). While PEDOT is a conducting polymer with high stability in aqueous solution, the amphiphilic nature of poly(sodium-4-styrenesulfonate) (NaPSS) allowed repelling of the cresol oxidation products thus reducing electrode fouling. After 20 repetitive measurements, the measured currents at the modified and bare GCE were 85% and 30% of the initial value, respectively. The modified sensor exhibited a linear range between 50 and 300 ppb and allowed continuous monitoring of TCP without requiring electrode polishing that may limit its practical applicability in the aircraft cabin.

### 2.3. Zwitterionic Polymers

Sun et al. have reported very recently a method to fabricate antifouling microarrays for protein sensing through photopolymerization of biomimetic betaine compounds [29]. The microarrays involved functionalizable polycarboxybetaine methacrylate (pCBMA)-grafted arrays and a nonfunctionalizable polysulfobetaine methacrylate (pSBMA)-grafted background. The new type of microarray was modified with abundant carboxyl groups and zwitterionic polymers allowing efficient immobilization of capture antibodies against bovine serum albumin (anti-BSA) and the detection of low biomolecule concentration (10 ng mL^−1^ BSA) with excellent antifouling properties in complex matrices (100% bovine serum). 

### 2.4. pH-Responsive Methacrylate Polymers

Wang´s group reported very recently an attractive strategy for addressing the biofouling challenge of electrochemical biosensors. They used commercial methacrylate polymeric coatings (Eudragit, Evonik Industries, polymers) with pH-responsive behavior. Rather than protecting the sensor during the operation in complex biofluids, the reported strategy relied on turning “ON” different sensors, at sequential preselected times, thus ensuring no biofouling even after prolonged contact with biological media. For this operation, transient methacrylate-based coatings were used, with different dissolution times at specific pH values, leading to delayed exposure of the fresh transducer surface. This interesting strategy has been exploited to develop electrochemical sensors by coating bare carbon or glucose oxidase (GOx)-Prussian Blue (PB)-graphite screen-printed electrodes with Eudragit^®^ L100 [30]. Carbon paste biosensors prepared from edible materials such as olive oil and activated charcoal have demonstrated to protect the activity of the embedded GOx by coating the electrode surface with pH-responsive polymers which dissolve below pH 5.0 (Eudragit^®^ E PO) and above pH 6.0 (Eudragit^®^ L100), and facilitate tuning the sensor activation in gastric and intestinal fluids, respectively, at specific predetermined times [31]. The excellent antibiofouling properties and tunable delayed actuation, simply by varying the type, density, and thickness of the coating, allowed direct glucose monitoring in raw undiluted blood and saliva samples [30] as well as in gastrointestinal (GI) fluids [31] over prolonged periods. While 100% of the initial response was obtained for glucose with the coated GOx-PB-graphite screen-printed electrodes after 2 h incubation in undiluted human saliva or blood samples, the uncoated biosensors provided just 50% of the initial signals [30].

## 3. Antibiofouling Hydrogels

Hydrogels are water swellable three-dimensional structures formed by chemical (covalent bonds) or physical (noncovalent interactions) cross-linking. These smart materials have excellent biocompatibility and great ability of water absorption and have been proposed as excellent electrode modifiers to impart antifouling properties. Apart from this, hydrogels show other features which make them particularly interesting for electrochemical biosensing, including interaction with biological components at the molecular level, regulating viscoelastic properties, reaction to external stimuli, and the possibility to incorporate bioreceptors into their highly wet structure using a wide range of well-known synthesis methods [32]. Particularly interesting are electroconductive hydrogels—such as polypyrrole, polyaniline, and poly (ethylenedioxy thiophene)—in which conductive polymers facilitate electron transport across the interface and the porous hydrogels provide a large surface area with greater diffusivity [32].

A three-dimensional nanocomposite composed of a gold nanoparticle (AuNP)-loaded polypyrrole hydrogel was deposited on a GCE and successfully employed for the fabrication of a sensitive immunosensor for label-free determination of carcinoembryonic antigen (CEA) [33]. The immunosensor offered a wide linear detection range (1 fg mL^−1^–200 ng mL^−1^), a LOD of 0.16 fg mL^−1^, good selectivity, and satisfactory accuracy for the determination of CEA in human serum samples.

**Figure 2:**
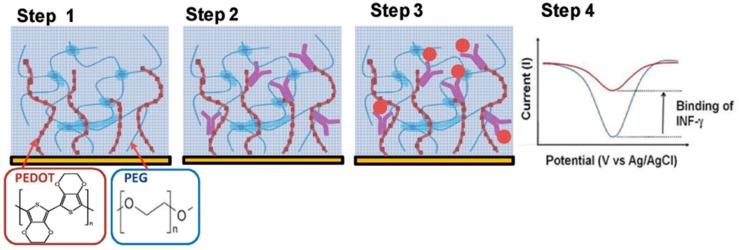
Preparation of an immunosensor for B-IFN-γ using poly (3,4-ethylenedioxythiophene) poly (ethylene glycol) (PEDOT/PEG)-conductive hydrogels. (**Step 1**) Fabrication of the PEDOT/PEG nanocomposite hydrogel forming the PEG gel film on the electrode surface and electropolymerizing the PEDOT on the PEG gel. (**Step 2**) incorporation of specific monoclonal antibodies into the gel via PEDOT-COOH groups. (**Step 3**) selective immunorecognition of the target B-IFN-γ. (**Step 4**) Comparison of the PEDOT reduction peak current measured by CV before (blue) and after (red) B-IFN-γ binding. Reproduced from [34] with permission.

Shin et al. [34] reported the combined use of PEG hydrogels with PEDOT to prepare conductive and antifouling electrode coatings. The PEDOT/PEG nanocomposite hydrogel was used to develop an electrochemical immunosensor to determine bovine-interferon-γ (B-IFN-γ) involving covalent immobilization of specific monoclonal antibodies using 1-ethyl-3-(3-dimethylaminopropyl)-carbodiimide/N-hydroxysuccinimide (EDC/NHS) chemistry onto the PEDOT-COOH groups. As it is shown in Figure 2 (step 4), the antigen binding provoked a decrease in the intrinsic PEDOT peak reduction current measured by cyclic voltammetry (CV). This coating showed nonfouling properties thus allowing operation in whole bovine blood with a LOD of 1 ng mL^−1^ IFN-γ compared to 0.5 ng mL^−1^ achieved in pristine buffer. The immunosensor was applied to monitoring of IFN-γ released from bovine leukocytes.

## 4. Antibiofouling Peptides and Peptoid-Based Materials

Naturally occurring biomolecules such as amino acids, peptides, and polysaccharides, thought to undergo structural improvements under physiological conditions to prevent biofouling, are also used in the development of innovative antifouling approaches [7,35]. 

Peptides are prospective candidates among antifouling materials because, apart from their ease design and synthesis, they are composed of natural amino acids which are zwitterionic molecules in biological systems and, therefore, possess inherently outstanding biocompatibility [36]. Besides superior biocompatibility and coordination ability, particularly designed peptides with strong hydrophilicity and neutral charge [37,38,39] help to prevent nonspecific protein adsorption through hydrophobic interaction and charge attraction, respectively [5,6].

On the other hand, peptoids are non-natural biomimetic polymers which, unlike PEG and zwitterions and similarly to peptides, allow tuning their surface structure and antifouling ability [7,35,40]. The sequence specificity of polypeptoids enables incorporating at precisely known locations known antifouling functionalities using a single backbone chemistry which allows envisioning the design of libraries with sequence-specific materials for optimum performance [7].

**Figure 3:**
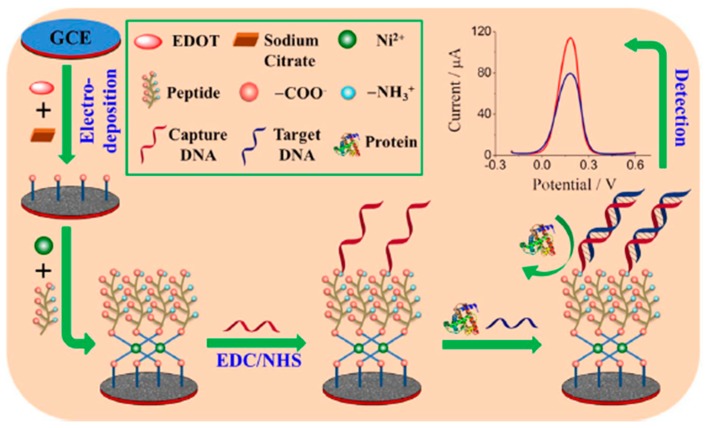
Electrochemical DNA sensor for *BRCA1* determination involving the use of a zwitterionic peptide anchored to the conducting polymer PEDOT. Reproduced from [41] with permission.

Zwitterionic peptides terminated with carboxylic groups, anchored to a conducting polymer of citrate-doped PEDOT via nickel cation coordination, with remarkable antifouling ability and good conductivity, were proposed for the construction of an electrochemical DNA sensor for the determination of the *BRCA1* breast cancer biomarker [41]. A layer of the conducting polymer PEDOT was electrodeposited on a GCE in a solution containing 3,4-ethylenedioxythiophene (EDOT) and sodium citrate. During the deposition process, citrate ions with three negatively charged carboxyl groups were incorporated into the formed PEDOT polymer as dopant. Some of the carboxyl groups on the surface were used for the subsequent immobilization of peptide which, in turn, was used for the immobilization of the carboxyl group-terminated *BRCA1* recognition probe through the formation of amide bonds (Figure 3). The probe/target DNA hybridization event was monitored by the decrease in the DPV signal measured in the presence of [Fe(CN)_6_]^3−/4−^. The DNA biosensor achieved a LOD value of 0.03 fM and was employed to perform the determination of the target DNA in doped 5% (*v*/*v*) human plasma with a satisfactory accuracy.

**Figure 4:**
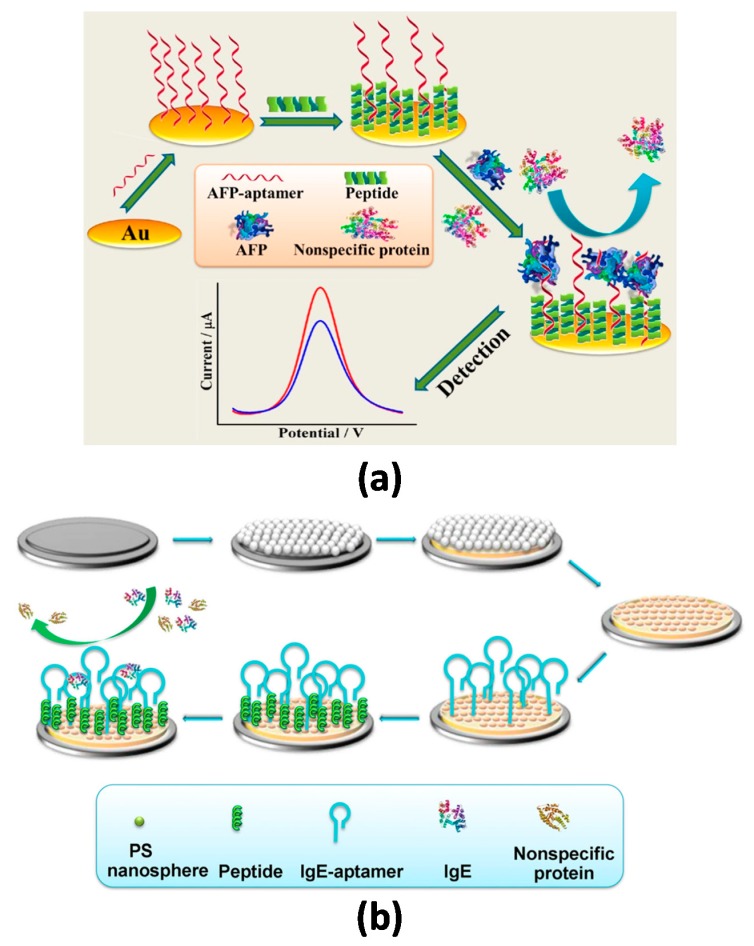
Schematic illustration of antifouling aptasensing interfaces developed through self-assembly of zwitterionic peptides and the specific aptamers for (**a**) alpha-fetoprotein (AFP) determination onto a gold electrode and (**b**) IgE onto a macroporous Au substrate electrochemically fabricated on a GCE. Reprinted from (a) [6] and (b) [5] with permission.

Cui et al. [6] reported a low-fouling aptasensor for the determination of alpha-fetoprotein (AFP) using mixed self-assembled aptamers and newly designed zwitterionic peptides. The long-chained AFP-specific aptamers acted as the recognizing layer and the designed short-chained zwitterionic peptides (with good hydrophilicity, nearly electrical neutrality) endowed biocompatibility and antifouling ability against nonspecific protein adsorption. The bioreagents were immobilized successively onto a gold electrode via Au–S bonding (Figure 4). By measuring the DPV responses of the sensor before and after target binding in the presence of [Fe(CN)_6_]^3−/4−^, a linear correlation with the logarithmic value of AFP concentration was observed between 10.0 fg mL^−1^ and 100.0 pg mL^−1^ with a LOD value of 3.1 fg mL^−1^. Importantly, the biosensor showed satisfying antifouling ability after 30 min incubation in 1−2% serum and was successfully applied to the analysis of 1/10,000 (*v*/*v*) diluted human serum samples. The same group has recently proposed an antifouling aptasensing interface for immunoglobulin E (IgE) through mixed self-assembly of thiolated zwitterionic peptide/specific aptamer onto a macroporous Au substrate electrochemically fabricated with the aid of multilayer polystyrene nanospheres self-assembled on a GCE [5]. The aptasensor exhibited an excellent sensitivity (linear range of 0.1 to 10 pg mL^−1^ and LOD of 42 fg mL^−1^) and successful performance in 5% (*v*/*v*) bovine serum without significant biofouling.

## 5. Antibiofouling Thiolated Monolayers

**Figure 5:**
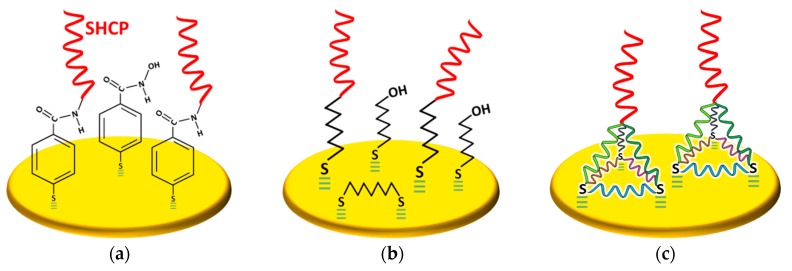
Schematic illustration of three different antifouling thiolated monolayers: (**a**) thioaromatic DNA monolayer prepared by covalent immobilization of amino-functionalized capture probes onto *p*-mercaptobenzoic acid (*p*-MBA) monolayers; (**b**) ternary DNA monolayer composed of a SHCP, 1,6-hexanedithiol (HDT) and MCH; and (**c**) thiolated DNA tetrahedral nanostructures.

SAMs of thiol-tethered DNA probes on Au electrodes surfaces have been widely used to construct nucleic acid sensing bioplatforms with improved flexibility and stability [42,43,44]. In this context, it is well known that electrode modification with thiolated capture probes (SHCPs) provides poor target nucleic acid hybridization [45] due both to the undesirable lying down conformation of the probes, immobilized through the nitrogenous bases to the gold surface, and/or their high packing. Therefore, to control the density, displace off the gold surface any weakly-adsorbed probe and stand up the immobilized SHCPs on the electrode surface to ensure an optimal hybridization with the target, a second alcohol-terminated diluent thiol component is frequently post-assembled to the surface which also prevents unspecific adsorptions due to the polar −OH head groups. Apart from the most widely used mercaptohexanol (MCH), 2-mercaptoethanol, 4-mercaptobutan-1-ol, 2-mercaptoundecanoic acid, and 11-mercaptoundecanol have also been employed for such purpose [42,45,46,47,48,49]. However, SHCP/MCH binary monolayers still display nonspecific background contributions, particularly from proteins [50], due to incomplete backfilling and irreproducibility issues during the assembling (attributed to the presence of surface defects and lateral diffusion of the SHCPs triggered after MCH backfilling) which have a negative impact on the hybridization efficiency and limit their long-term stability [42,44,48,49,51,52,53,54,55,56]. Nevertheless, a rational design of the surface chemistry involving the use of thioaromatic DNA monolayers (Figure 5a), ternary monolayers (Figure 5b) or tetrahedral DNA nanostructures (Figure 5c) has demonstrated allowing to control the spacing between the attached probes and minimizing nonspecific adsorptions endowing the modified interfaces with better storage stability and sensing performance in complex samples [57,58,59,60]. 

### 5.1. Thioaromatic DNA Monolayers

SAMs of aromatic thiols compare advantageously with their aliphatic counterparts in antifouling properties, packing efficiency, high electrical conductivity due to the delocalized π electrons in the aromatic phenyl ring [56], and strong structural stiffness [61,62]. In these thioaromatic layers, the intermolecular rather than the head group–gold interactions, are the key factors determining packing which lead to a considerable improvement of surface quality and nonfouling properties. 

Lobo-Castañón´s research group exploited the use of thioaromatic DNA monolayers for the fabrication of DNA sensors constructed on thin gold films in connection with amplification strategies such as helicase-dependent amplification (HDA) [63] or at commercial gold screen-printed electrodes without amplification [55]. The biosensors were applied to the detection of relevant nucleic acids in genetically modified organisms (Cauliflower Mosaic Virus 35S Promoter-specific DNA sequence) [63] and *Legionella pneumophila* (16S ribosomal RNA, rRNA) [55]. 

Binary thioaromatic SAMs were prepared by using the aromatic thiol *p*-aminothiophenol (*p*-ATP) instead of MCH as diluent in DNA self-assembled monolayers (SHCP+*p*-ATP SAM) [55] or by incorporating SHCP into *p*-ATP monolayers previously subjected to potential cycling at acidic pH through the so-called inserting method (*p*-ATP+SHCP SAM) [63]. Depending on the gold substrate type, the use of *p*-ATP as a backfilling agent instead of MCH led to decreases between 6 and 16 times in the background current [56]. 

The same authors also proposed the use of *p*-MBA monolayers as scaffolds to covalently immobilize amino-functionalized capture probes (Figure 5a) [55]. An exhaustive comparison between the sensing performance provided by the three types of thioaromatic monolayers showed that the LOD achieved for the determination of the same short synthetic DNA target followed the order: *p*-ATP+DNA (6 pM) < DNA-*p*-MBA (40 pM) < DNA+*p*-ATP (200 pM). However, the determination of the long target native RNA extracted from *Legionella pneumophila* showed the trend DNA-*p*-MBA (110 pM) < *p*-ATP+DNA (1.2 nM) [55]. These findings suggested that pure thioaromatic monolayers avoided the formation of DNA clusters due to the efficient blocking ability of the aromatic thiol and achieved better hybridization efficiency, particularly for high length nucleic acid targets such as bacterial rRNA, than electrodes modified with the binary thioaromatic SAMs because of *p*-MBA acted as a rigid spacer maximizing the target accessibility to the immobilized capture probe [55,56]. These DNA biosensors demonstrated successful applicability for determining *Legionella pneumophila* directly in purified 16S rRNA extracted without previous amplification.

### 5.2. Ternary DNA Monolayers

Ternary SAMs have been used for the preparation of nucleic acid-based biosensors [44,52,53,64,65,66,67,68] and aptasensors [69] and involve the self-assembling of two thiolated compounds as diluents/spacers together with an appropriate SHCP on gold electrodes. The third component can be a charged component such as 3-mercaptopropionic acid (MPA) [51,70,71] or a cyclic [52] or linear [53,58,72,73] dithiol. 

Exhaustive optimization studies performed with ternary monolayers demonstrated that the fabrication protocol, nature, and concentration of the functional groups; chain length of the third component [44,53]; and type/roughness of the gold electrode [74] are key parameters in affecting the hybridization efficiency, surface coverage, and nonspecific adsorptions of the resulting biosensors. 

Dharuman et al. [51,70,71] were pioneers in demonstrating that ternary mixed monolayers prepared by sequential self-assembly of SHCPs, and the use of MCH and MPA as diluents allowed controlling both probe orientation and surface passivation, leading to higher hybridization and discrimination efficiencies attributed to the optimal spacing between ssDNA probes. Due to the MPA-ssDNA hydrophilic interactions instead of MCH-ssDNA hydrophobic interactions, MPA demonstrated to be more efficient than MCH both in minimizing nonspecific adsorptions and placing the SHCPs perpendicularly to the electrode surface [51]. Moreover, each MPA molecule binds two MCH molecules generating hydrogen bonds and forming a pseudo-layer which decreased the unspecific adsorptions [44,71]. 

Wang´s research group proposed in a pioneering way the use of ternary DNA SAM-interfaces prepared by co-immobilization of a short cyclic [52] or linear [53,64,65,66,67,68,69] dithiol with the SHCP, followed by chemisorption of MCH in a subsequent step. These ternary SAMs provided a significant reduction in the background current and 100-fold improvements in the signal-to-noise (S/N) ratios [52]. Thus, improved behavior was attributed to an efficient passivation of the free gold surface, by the lying-flat self-assembled dithiol covering these unspecific adsorption points, without sacrificing the electron transfer through the biosensing layer. Among the different evaluated dithiols—DL-dithiothreitol (DTT), 1,3-propanedithiol (PDT), HDT (Figure 5b), and 1,9-nonanedithiol (NDT)—the ternary layers prepared with linear dithiols with 3–6 carbon atoms in length exhibited a better analytical performance (larger S/N). This was attributed to the preferential lying-down configuration adopted by these linear dithiols in the experimental conditions assayed, which minimized nonspecific adsorptions while maintained larger amount of immobilized capture probe compared to the NDT and allowed better permeability of small signaling molecules than the compact layers formed in the presence of DTT.

These ternary monolayers, assembled onto 16-Au electrode arrays prepared by photolithography [52,53,65,66,67,68], Au interdigitated electrode arrays [69] and screen-printed Au electrodes [64], were employed in connection with DNA hybridization biosensors for the improved determination of bacterial rRNA [52,53,64,65,67,68] peanut allergen and gluten-encoding DNA sequences [44] and precursor rRNAs [66] without target nucleic acid amplification and showing remarkable fouling resistance in raw bacterial lysates and undiluted human serum and urine. Ternary monolayers were also used to prepare aptasensors for the impedimetric determination of proteins such as IFN-γ [69] and thrombin [74].

**Figure 6:**
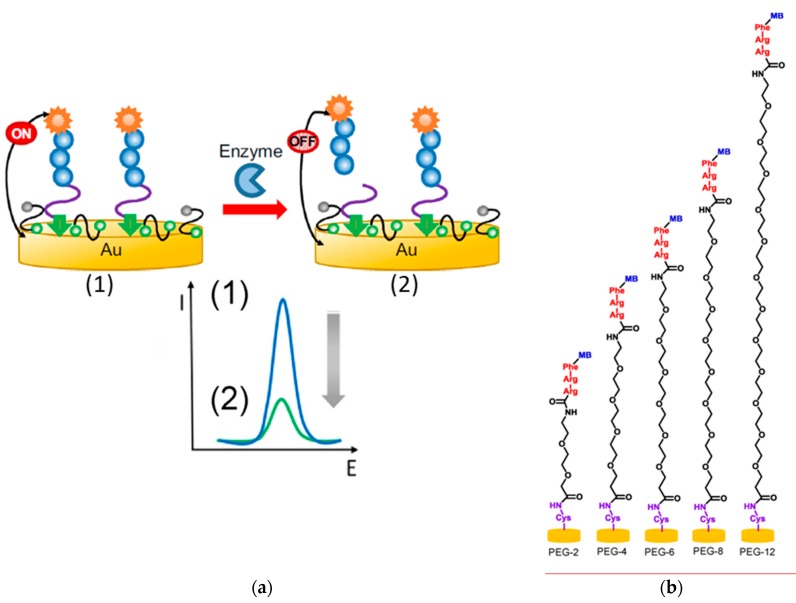
Fundamentals of the peptide-based electrochemical platforms functioning. (**a**) The target protease catalyzes the cleavage of the immobilized redox-labeled peptide (1), releasing the redox-containing fragment into solution (2) leading to a decrease of the SWV electrochemical signal. (**b**) Chemical structures of the different PEG spacer lengths evaluated in the redox-labeled peptide probe (number of EG units are denoted by PEG-x). Reproduced from (**a**) [72] and (**b**) [58] with permission.

Bradley´s research group proposed the use of ternary SAMs composed of a redox-tagged peptide, MCH, and a linear dithiol, PEG [72] or PDT [58,73] to construct reagent-free integrated aptasensors with minimal nonspecific adsorptions for the detection of the activity of relevant proteases. The reported strategies involved the use of PEG as dithiol [72] (Figure 6a) or as a spacer in the peptide sequence [58] (Figure 6b) of the multicomponent SAMs. The detection principle relied on the specific proteolytic cleavage of the redox-tagged peptides by the target protease, trypsin [58,72] or human neutrophil elastase (HNE, related with polymorphonuclear neutrophil activity [73]), which provoked the release of the redox reporter (MB), resulting in a decrease of its peak current measured by square wave voltammetry (SWV). The use of PEG spacer was shown to be important in tuning both the antifouling properties and the probe’s flexibility [58]. The peptide-based sensors provided LODs of 88–250 pM [58,72] for trypsin and 4 nM for HNE [73] and were applied to determine the target proteases in human blood.

### 5.3. PEG-based SAMs 

Henry et al. [75] compared the performance of three different PEG co-immobilization strategies in the preparation of DNA sensors for the detection of the breast cancer marker estrogen receptor-α (*ESR1*). PEGylated *ESR1* DNA capture probes were co-immobilized in the presence of either a PEG alkanethiol (thiolated hexadacanetriethyelene glycol), a mixture of PEG alkanethiol and MCH or a bipodal aromatic PEG alkanethiol (dithiolated aromatic triethylene glycol) onto an electrode array chip consisting of 16 square-shaped gold electrodes arrayed in a 4×4 configuration with a geometrical area per electrode of 1 mm^2^. The strategy, consisting of co-assembled thiolated *ESR1* capture probes and bipodal aromatic PEG alkanethiol at a ratio of 1:100, provided better performance in terms of sensitivity and minimal nonspecific binding. The method was applied to the analysis of the product resulting from the polymerase chain reaction (PCR) amplification of the genetic material extracted from breast cancer cells (MCF-7) and detected 0.17 nM of the synthetic amplicon. 

### 5.4. Tetrahedral DNA Nanostructures 

Pyramidal tetrahedral DNA nanostructures are reproducible self-assembled onto gold surfaces by using four carefully designed ss-DNA probes (three of them thiolated) which constitute the six edges of a DNA tetrahedron and leave pendant a linear sequence at the top of the bound tetrahedron (Figure 5c) [76,77]. The self-assembling one-step process is simple, rapid (within 2 min), has high yield (over 85%), and leads to rigid, stable, and reproducible bioscaffolds suitable for attaching biorecognition elements in an upright orientation and far away from the electrode surface. This solution-phase-like environment imparts optimal efficiency in the affinity reactions and excellent antifouling properties without requirement of a posterior backfilling step [59,60,78,79,80,81]. The resulting nucleic acid biosensing scaffolds were able to tailor sensitivity according with the required application by the precise control of the spacing between the immobilized probes [82], exhibited higher stability (the tetrahedron moved slower than monothiolated probes [77]), a 5000-fold greater affinity, and were less-prone to unspecific adsorptions than the biosensors constructed with single point-tethered oligonucleotides [83]. The tetrahedral bioscaffolds were used to immobilize DNA probes [77,79,82,83,84,85], antibodies [80,86,87], and aptamers [77,78] as well as in connection with different amplification strategies including hybridization chain reaction (HCR [79]), rolling circle amplification (RCA [88]), analyte-triggered nanoparticle localization-HCR dual amplification [89], DNA tetrahedral nanostructures as reporter probes [85], and guanine nanowire amplification [90]. These low fouling DNA nanostructured bioplatforms were applied to the determination of nucleic acids (DNAs [77,82,83,84] and miRNAs [79,88,89,90,91,92,93]) proteins (TNF-α [86], thrombin [77], and prostate specific antigen (PSA) [80]) and peptides (pneumococcal surface protein A (PspA) peptide [87]) as well as of small molecules (cocaine [78]) in particularly fouling samples such as serum [77,78,80,82,90], PCR products from clinical samples [83], cell lysates [87], and total RNA extracted from cells [88,89], serum [88], and tumor tissues [91,92].

### 5.5. Other Thiolated SAMs 

Goda et al. [12] reported the one-step synthesis of phosphobetainetype alkanethiolate from 2-methacryloyloxyethyl phosphorylcholine (MPC) as a building block to prepare an antifouling SAM. These authors functionalized one mercapto group in 1,6-hexanedithiol with MPC through the Michael type addition between methacrylate and thiol groups under mild conditions. The remaining mercapto group in MPC-SH was chemisorbed onto the gold surface to prepare antifouling SAMs against proteins and cells. 

McQuistan et al. [94] proposed the incorporation of short thiolated oligonucleotides as passivating diluents in the fabrication of electrochemical peptide-based (E-PB) sensors for the determination of autoantibodies against HIV-1 p24 antigen with alleviating fouling properties. The method formed a negatively charged layer capable of resisting nonspecific adsorption of matrix contaminants. DNA diluents containing four thymine (T) bases connected to a 6-carbon alkanethiol linker provided better performance and determined 70 nM of target IgG in 10% human saliva samples. 

**Figure 7:**
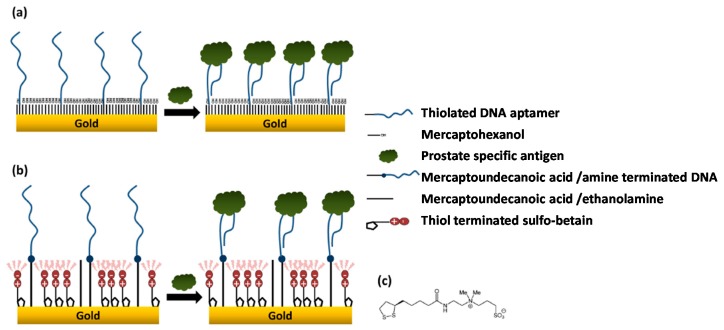
Aptasensors prepared for PSA determinations using (**a**) conventional binary self-assembled monolayers (SAMs) composed of a thiolated specific aptamer and mercaptohexanol (MCH) and (**b**) a SAM prepared by covalent immobilization on the amine terminated aptamer onto 11-mercaptoundecanoic acid and thiolated sulfobetaine (structure displayed in (**c**)). Reproduced from [95] with permission.

Jolly et al. [95] proposed a novel binary SAM to construct a label-free aptasensor for the determination of PSA. They compared the performance provided by aptasensors prepared by assembling the conventional binary SAMs composed of MCH and thiolated-DNA aptamer on a polycrystalline gold surface or the new layer involving 11-mercaptoundecanoic acid for further covalent immobilization of amine terminated DNA aptamers and sulfobetaine terminated thiol as an antifouling agent (Figure 7). The electrodes modified with this latter layer could detect 1000 times lower PSA levels (1 ng mL^−1^ vs 10 µg mL^−1^) which falls in the serum clinically relevant range (1–10 ng mL^−1^) and also prevented nonspecific binding of the human serum albumin used as control protein, compared to the high nonspecific binding observed at the MCH-based sensors. 

## 6. General Conclusions and Personal Viewpoints

It is unquestionable that despite the tremendous possibilities and capabilities provided by electrochemical biosensors for the determination of relevant analytes in different fields, the requirements involved in the determination of fouling analytes, such as phenols and neurotransmitters, of target analytes directly in samples rich in proteins, with extreme pH values (denaturing the biorecognition elements), or after prolonged incubation periods of the biosensors in this type of matrices are important challenges yet to be faced up.

With this purpose, efficient electrode modification strategies with a wide range of antifouling (bio)materials (polymers, hydrogels, peptides, and thiolated monolayers) have been critically discussed in this review. These strategies have addressed severe electrode (bio)fouling processes and have allowed the preparation of biosensors able to perform the electrochemical determination of fouling analytes or in fouling samples with excellent performance. Although a comparison with the use of nonbiological antifouling nanomaterials is out of the scope of this review, as far as we know, their properties have not been thoroughly compared under identical conditions. In principle, the use of biomaterials should be advantageous mainly in terms of reproducibility, simplicity, biocompatibility and versatility. Additionally, as antifouling (bio)materials have been used in specific areas, it is difficult to make a general choice among them as more adequate for real applications and even to carry out an exhaustive comparison of their performance. Nevertheless, the recently reported use of commercial methacrylate polymeric coatings appears as a particularly interesting antifouling strategy in terms of biocompatibility, simplicity, preparation time, and versatility to be further explored to allow continuous electrochemical sensing and biosensing in real-world applications regardless composition and pH of the sample.

However, given the diversity of electrode fouling analytes, the coexistence of different fouling agents in the same sample and the wide variety of fouling samples where relevant analytes should be analyzed, more research on antifouling strategies is required to minimize the significant impact of electrode fouling and ensure their appropriate functioning in harsh environments. 

In addition, currently reported antifouling approaches should be evaluated against a wider variety of fouling agents and matrices, particularly biological samples and matrices that are a complex mixture of proteins, peptides, lipids, and carbohydrates. Nevertheless, new strategies should be developed to overcome constraints or improve the performance of the existing ones. Moreover, it is worth to mention that in many cases the fouling resistance of a given strategy is evaluated only towards one analyte or a particular matrix sample which limits a general assessment of how the antifouling strategy may work in other environments or over other electrochemical substrates. This lack of information makes not straightforward to extrapolate the particular results to select a priori a strategy able to meet the demands for a particular application. In this context, the use of computational approaches, such as the Quantitative Structure–Property Relationship (QSPR), may help tremendously the prediction of the behavior of (bio)materials under different environments as well as the design of in silico strategies able to meet the required antifouling demands, thus saving a lot of experimental work. 

Therefore, future efforts must be aimed at evaluating antifouling strategies against different concentrations of the same fouling agent (fouling may happen with several antifouling approaches only above a certain concentration) and with a wide variety of fouling agents and matrices (blood, serum, urine, saliva) to develop strategies with broad applicability. 

Most antifouling strategies involve modification of the electrode surface with a coating or film to impart fouling resistance. The modifier choice depends on the analyte, conditions of the system, and the type of electrode. However, the pathways involved in electrode fouling and the mechanisms by which antifouling strategies can minimize or eliminate fouling are generally poorly understood. Therefore, research should be driven also to advance in that knowledge aiming to develop efficient and versatile antifouling strategies able to address unmet challenges and extend the range of applications. The active pursuit of new strategies to improve the already remarkable antifouling properties imparted by current strategies is encouraging and envisions better materials to be used in a near future. New materials with unique or improved properties will lead to develop highly stable electrochemical biosensing systems for continuous on-body determination or in vivo monitoring of important analytes directly in a variety of body fluids of different pH range and composition.

It is important to note that these antifouling (bio)material-based electrochemical sensors have been reported so far as prototypes and tested only under laboratory conditions. Therefore, their commercialization is still far away. The development of versatile strategies by a better understanding of the antifouling mechanisms will allow their application under different conditions. The optimization of storage stability and transportation conditions of the antifouling (bio)material-based electrochemical sensors are also important challenges to face up for constructing marketable devices with reliable and proper functioning in different environments.

## Abbreviations

AFP	alphafetoprotein
*p*-ATP	*p*-aminothiophenol
AuNPs	gold nanoparticles
B-IFN-γ	bovine-interferon-γ
BSA	bovine serum albumin
CEA	carcinoembryonic antigen
CV	cyclic voltammetry
DNA	deoxyribonucleic acid
DPV	differential pulse voltammetry
DTT	DL-dithiothreitol
EDC	1-ethyl-3-(3-dimethylaminopropyl)-carbodiimide
EDOT	3,4-ethylenedioxythiophene
E-PB	electrochemical peptide-based
GI	gastrointestinal
GCE	glassy carbon electrode
GOx	glucose oxidase
HDA	helicase-dependent amplification
HDT	1,6-hexanedithiol
HNE	human neutrophil elastase
IgE	immunoglobulin E
LOD	limit of detection
MB	methylene blue
*p*-MBA	*p*-mercaptobenzoic acid
MCH	mercaptohexanol
MEA	monoethanolamine
MPA	3-mercaptopropionic acid
MPC	2-methacryloyloxyethyl phosphorylcholine
NaPSS	poly(sodium-4-styrenesulfonate)
NDT	1,9-nonanedithiol
NHS	N-hydroxysuccinimide
PANI	polyaniline
pCBMA	polycarboxybetaine methacrylate
PCR	polymerase chain reaction
PDT	1,3-propanedithiol
PEDOT:PSS	poly(3,4-ethylenedioxythiophene)-poly(styrene sulfonate)
PEG	poly(ethylene glycol)
PB	prussian blue
PSA	prostate specific antigen
pSBMA	polysulfobetaine methacrylate
PspA	pneumococcal surface protein A
QSPR	Quantitative Structure-Property Relationship
RNA	ribonucleic acid
rRNA	ribosomal RNA
SAMs	self-assembled monolayers
SHCP	thiolated capture probe
S/N	signal-to-noise
ssDNA	single-stranded DNA
SWV	square wave voltammetry
TCP	tricresyl phosphate
